# Molecular epidemiology of clinical *Mycobacterium tuberculosis* complex isolates in South Omo, Southern Ethiopia

**DOI:** 10.1186/s12879-020-05394-9

**Published:** 2020-10-13

**Authors:** Biniam Wondale, Kwon Keehwan, Girmay Medhin, Takele Teklu, Temesgen Mohammed, Samuel Tolosa, Aboma Zewude, Friehiwot Amsalu, Rembert Pieper, Gobena Ameni

**Affiliations:** 1 Aklilu Lemma Institute of Pathobiology, Addis Ababa University, PO. Box 1176, Addis Ababa, Ethiopia; 2 Department of Biology, Arba Minch University, PO. Box 21, Arba Minch, Ethiopia; 3 J. Craig Venter Institute, Rockville, MD USA; 4 Department of Immunology and Molecular Biology, University of Gondar, PO. Box 196, Gondar, Ethiopia; 5 Ethiopian Public Health Institute, P.O.Box 1242, Addis Ababa, Ethiopia; 6 Jinka General Hospital, Jinka, Ethiopia; 7 Department of General Surgery, College of Medicine and Health Sciences, University of Gondar, Gondar, Ethiopia; 8 Department of Veterinary Medicine, College of Food and Agriculture, United Arab Emirates University, P.O.Box 15551, Al Ain, UAE

**Keywords:** *M. Tuberculosis*, Molecular epidemiology, Spoligotyping, MIRU-VNTR, South Omo

## Abstract

**Background:**

Tuberculosis (TB) is caused by *Mycobacterium tuberculosis* complex (MTBC). Mapping the genetic diversity of MTBC in high TB burden country like Ethiopia is important to understand principles of the disease transmission and to strengthen the regional TB control program. The aim of this study was to investigate the genetic diversity of *Mycobacterium tuberculosis* complex (MTBC) isolates circulating in the South Omo, southern Ethiopia.

**Methods:**

MTBC isolates (*N* = 156) were genetically analyzed using spacer oligotyping (spoligotyping) and mycobacterial interspersed repetitive unit-variable number of tandem repeat (MIRU-VNTR) typing. Major lineages and lineages were identified using MTBC databases. Logistic regression was used to correlate patient characteristics with strain clustering.

**Results:**

The study identified Euro-American (EA), East-African-Indian (EAI), Indo-Oceanic (IO), Lineage_7/Aethiops vertus, *Mycobacterium bovis* and *Mycobacterium africanum* major lineages in proportions of 67.3% (105/156), 22.4% (35/156), 6.4% (10/156), 1.9% (3/156), 1.3% (2/156) and 0.6% (1/156), respectively. Lineages identified were Delhi/CAS 23.9% (37/155), Ethiopia_2 20.6% (32/155), Haarlem 14.2% (22/155), URAL 14.2%(22/155), Ethiopia_3 8.4% (13/155), TUR 6.5% (10/155), Lineage_7/Aethiops vertus 1.9% (3/155), Bovis 1.3% (2/155), LAM 1.3% (2/155), EAI 0.6% (1/155), X 0.6% (1/155) and Ethiopia H_37_Rv-like strain 0.6% (1/155). Of the genotyped isolates 5.8% (9/155) remained unassigned. The recent transmission index (RTI) was 3.9%. Orphan strains compared to shared types (AOR: 0.09, 95% CI: 0.04–0.25) were associated with reduced odds of clustering. The dominant TB lineage in pastoral areas was EAI and in non-pastoral areas was EA.

**Conclusion:**

The epidemiological data, highly diverse MTBC strains and a low RTI in South Omo, provide information contributing to the TB Control Program of the country.

## Background

The *Mycobacterium tuberculosis* complex (MTBC) constitutes a group of mycobacteria which are 99.9% similar at the nucleotide level and the causative agents for tuberculosis (TB) [11]. Globally, TB became the leading cause of death from an infectious disease [39]. Ethiopia stands 12th in the world and 4th in Africa among the high TB burden countries with 24,000 TB deaths and 165,000 new TB cases in 2018 [39]. The current prevalence of MDR/RR-TB in Ethiopia is 0.71 and 16% in new and previously treated TB cases, respectively [39].

Understanding the molecular epidemiology of TB is important for regional disease control. For instance, distinct strains may be linked to outbreaks [10], high virulence [42], emergency of drug resistance [44], disease progression [43], and can point to the geographic origin of a strain [20, 33] as well as identify new lineages [17, 28].

The South Omo Zone is an administrative unit in the southern Ethiopia bordering Kenya and South Sudan. The area is remote with a poor infrastructure and high population diversity with 16 different ethnic groups. Forty-two percent of South Omo’s residents including 15 ethnicities have a pastoral life style. The facilities for health care and education are underdeveloped especially in the pastoral regions [4]. A previous study suggested that the prevalence of TB among pastoralists is higher than in other socio-economic groups in Ethiopia [24]. In depth, high resolution molecular epidemiological surveys are required to characterize the diversity of MTBC isolates in this remote pastoral region precisely.

Beginning in the 1990s a number of molecular genotyping techniques have evolved to differentiate MTBC at the species and strain levels [22]. Whole genome sequencing is ideal to identify a strain type, but technically and informatically demanding and too expensive to characterize regional MTBC diversity [22]. Widely used in TB research are spacer oligotyping (spoligotyping) and mycobacterial interspersed repetitive units – variable numbers of tandem repeat (MIRU-VNTR). Spoligotyping targets a single locus and has less discriminatory power but is simple and cost effective. MIRU-VNTR targets numerous loci with increased discriminatory power. International databases and data analysis tools were created for both genotyping methods [22].

Most MTBC genotyping studies in Ethiopia employed spoligotyping [3, 6, 7, 16, 19, 26, 43]. We are aware of only very few Ethiopian studies using spoligotyping and MIRU-VNTR simultaneously to profile MTBC strains [1, 9, 36, 37, 43]. New lineages such as the MTBC lineage lineage_7/Aethiops vertus [17, 28], Ethiopia_2 and Ethiopia_3 [37] were newly assigned. Most surveys were in geographic areas more accessible than South Omo. The objective of the current study is to examine the MTBC population structures in the latter region and compare the data to those of other Ethiopian regions and globally.

## Methods

### Human subject recruitment and specimen collections

As part of a study to investigate TB in South Omo using both molecular epidemiology and systems biology methods between 2014 and 2017, we established a human subject protocol and a consent form to recruit more than 2000 individuals from different ethnic groups and administrative districts. We discerned non-pastoral areas (Debub Ari, Semen Ari and part of Male) and pastoral areas (Bena Tsemay, Hamer, Dassenech, Selamago and part of Male). The minimum age for enrolment was 15 years. According to 2007 population census, 577,673 people (7.5% urban and 92.5% rural) lived in the area with equal proportions of men and women [14]. Basic demographic and clinical data were collected using a pre-structured questionnaire.

Local translators explained the study’s objectives, risks and benefits to those individuals who were not able to communicate in the Amharic language. Those individuals who consented to participate in the study were asked to provide fine needle aspirate (FNA) and sputum samples to test for EPTB and PTB, respectively. Samples were stored at + 4 °C and -20 °C following collection and transferred to the sample processing and microbial culture laboratory in order to screen for growth of MTBC isolates. Individuals who were diagnosed with TB were educated on and received DOTS treatment according to the national TB management guideline [18].

### Generation of MTBC isolates

MTBC isolates were recovered from sputum and FNA samples for pulmonary TB (PTB) and extra-pulmonary TB (EPTB) cases, respectively. Mycobacterial cultures were performed at the Jinka Regional Laboratory (JRL). Samples were processed according to the modified Petroff’s method by decontaminating the specimen with an equal volume of 4% NaOH for 15 min; the remaining volume was filled with phosphate buffer saline (PBS) and centrifuged at speed of 3000 g for 15 min. A drop of phenol red was added to a pellet as pH indicator and neutralized using 10% HCl. The neutralized pellet was inoculated onto two LJ media. One supplemented with glycerol and the other with pyruvate. Inoculated media were incubated at 37 °C for up to 8 weeks. Mycobacterial growth was monitored every week. Culture was considered negative after 8 weeks if no growth was observed. Positive colonies were further confirmed by Ziehl-Neelsen (ZN) staining. Heat treatment of mycobacterial isolates in dH_2_O at 80 °C for 50 min was used for genomic DNA extraction without extensive DNA purification. Extracts were stored at -20 °C until they were used for molecular characterization.

### Molecular characterization

DNA of mycobacterial isolates were primarily subjected to region of difference (RD) 9 typing [11]. Secondly, a single tube amplification method [40] was applied for genus typing for isolates which were not detected by RD9 typing. Thirdly, spoligotyping was performed for MTBC isolates as described by Kamerbeek et al., [23]. Laboratory results of spoligotyping were interpreted in binary format and lineages were assigned using an updated version of the SITVITWEB [13] and major lineages were assigned using the “TB insight” database. Isolates which have similar patter to those in the SITVIT database were assigned a Spoligo International Type (SIT) number. Isolates not assigned to SIT numbers were referred as “Orphans” spoligotype. Finally, MTBC DNA samples were subjected to MIRU-VNTR typing following an established procedure [35]. Laboratory results of MIRU-VNTR typing were interpreted using the MIRU-VNTR*plus* database (http://www.miru-vntrplus.org) to determine MTBC strain lineages and relatedness [2, 38]. A minimum spanning tree (MST) was constructed. Previously identified Ethiopian strains were assigned manually to Ethiopia_1 (Lineage 7), Ethiopia_2 and Ethiopia_3, which lack the spoligotype based spacer 4–24, 13 and 10–19, respectively [9, 17, 37]. The RD9 typing, genus typing and spoligotyping were performed at Aklilu Lemma Institute of Pathobiology (ALIPB), Addis Ababa University (AAU), Addis Ababa, Ethiopia whereas MIRU-VNTR typing was performed at J. Craig Venter Institute (JCVI), Maryland, USA.

### Data analysis

Excel data were transformed into IBM SPSS version 20. The results were presented using descriptive statistics. The ability of genotyping methods to discriminate strains was calculated using Hunter-Gaston Discrimination Index (HGDI) [21]. The allelic diversity (*h*) based on MIRU-VNTR genotyping was calculated using HGDI and classified into relative discriminants based on previous [34] and newly proposed [27] index ranges. The recent transmission index (RTI) was calculated using a formula proposed by small et al. [33]. Isolates were said to be clustered if they have identical pattern based on spoligotyping and/or MIRU-VNTR. Clustering rate was determined using a formula (Nc-C)/N, where Nc = total number of isolates clustered, C = number of clusters, and N = total number of isolates [46]. The association between clustered strains and independent variables were computed using logistic regression models. The crude odds ratio (COR) and the adjusted odds ratio (AOR) were used to present the results. *P*-values of < 0.05 were considered statistically significant.

## Results

### Demographic and clinical characteristics of study participants

One thousand two hundred sputum and FNA samples of study participants were cultured for isolation of mycobacteria. Samples culture summary is presented in Table 1. In overall, 161 MTBC isolates were obtained upon RD9 and multiplex PCR based species and genus typing. Culture recovery rate vary from 2.5% (13/517) in community screened samples and 29.7% (33/111) in smear positive PTB samples from health facilities other than JGH to 67.3% (74/110) in smear positive PTB samples and 76.5% (26/34) in EPTB samples from JGH. Spoligotyping and MIRU-VNTR typing were carried out for a total of 156 isolates derived from 130 and 26 patients with clinical evidence of PTB and EPTB, respectively. Five samples were excluded due to insufficient quantities of DNA. Demographic and clinical characteristics of the corresponding 156 subjects are summarized in Table 2. The age range was 15–80 years with mean 32.9, median 30 and standard deviations 12.9. Female, PTB and new TB cases were 41.0% (64/156), 83.3% (130/156) and 95% (148/156), respectively.

**Table 1:** Culture summary of collected samples, South Omo, Ethiopia

	Samples from Health Facilities					Community > = 2 weeks cough in pastoral areas	Total
Source	JGH			Other than JGH^a^			
Sample type	Sputum Smear		FNA	Sputum Smear		Sputum	
	+ve	-ve		+ve	-ve		
Number of samples	110	404	34	111	24	517	1200
Recovered MTBC N (%)	74 (67.3)	14 (3.5)	26 (76.5)	33 (29.7)	1 (4.2)	13 (2.5)	161 (13.4)
Contamination N (%)	4 (3.6)	21 (5.2)	0 (0.0)	6 (5.4)	4 (16.7)	64 (12.4)	99 (8.3)

Abbreviations: *MTBC Mycobacterium tuberculosis* complex, *JGH* Jinka Genral Hospital; ^a^Other than JGH includes- Health centers in D/Ari, BenaTsemay, Dassenech, Hammer, Male, Gnagatom and Selamago

**Table 2:** Demographic and clinical characteristics of study participants in molecular epidemiology, South Omo, Ethiopia

Characteristics	Residential areas										Total N (%)
	Non-pastoral (***N*** = 70)			Pastoral (***N*** = 67)						Unspecified	
	D/Ari N (%)	Jinka N (%)	^a^Other areas N (%)	B/Tsemay N (%)	Dassenech N (%)	Hamer N (%)	Male N (%)	Gnangatom N (%)	Selemago N (%)		
Sex											
Female	23 (48.9)	6 (37.5)	1 (14.3)	6 (42.9)	4 (44.4)	4 (26.7)	5 (41.7)	1 (50.0)	7 (46.7)	7 (36.8)	64 (41.0)
Male	24 (51.1)	10 (62.5)	6 (85.7)	8 (57.1)	5 (55.6)	11 (73.3)	7 (58.3)	1 (50.0)	8 (53.3)	12 (63.2)	92 (59.0)
Age in years											
15–29	19 (40.4)	11 (68.8)	2 (28.6)	7 (50.0)	4 (44.4)	2 (13.3)	7 (58.3)	1 (50.0)	11 (73.3)	6 (31.6)	70 (44.9)
30–44	19 (40.4)	3 (18.8)	5 (71.4)	3 (21.4)	1 (11.1)	9 (60.0)	2 (16.7)	1 (50.0)	3 (20.0)	13 (68.4)	59 (37.8)
45+	9 (19.1)	2 (12.5)	0 (0.0)	4 (28.6)	4 (44.4)	4 (26.7)	3 (25.0)	0 (0.0)	1 (6.7)	0 (0.0)	27 (17.3)
Type of TB											
PTB	37 (78.7)	13 (81.2)	3 (42.9)	13 (92.9)	9 (100.0)	15 (100.0)	10 (83.3)	2 (100.0)	9 (60.0)	19 (100.0)	130 (83.3)
EPTB	10 (21.3)	3 (18.8)	4 (57.1)	1 (7.1)	0 (0.0)	0 (0.0)	2 (16.7)	0 (0.0)	6 (37.5)	0 (0.0)	26 (16.7)
Medical history											
New cases	45 (95.7)	16 (100.0)	7 (100.0)	12 (85.7)	9 (100.0)	12 (80.0)	12 (100.0)	2 (100.00)	14 (93.3)	19 (0.0)	148 (94.9)
Retreat. Cases	2 (4.3)	0 (0.0)	0 (0.0)	2 (14.3)	0 (0.0)	3 (20.0)	0 (0.0)	0 (0.0)	1 (6.7)	0 (0.0)	8 (5.1)
**Total**	47 (30.1)	16 (10.3)	7 (4.5)	14 (9.0)	9 (5.8)	15 (9.6)	12 (7.7)	2 (1.3)	15 (9.6)	19 (12.2)	156 (100.0)

^a^Other areas, include Addis Ababa, Konso, Semen Ari and Sawula; D/Ari, Debub Ari; B/Tsemay, Bena Tsemay; Retreat. Cases, anti-TB retreatment cases

### Discriminatory power of genotyping methods and MIRU-VNTR allelic diversity

All South Omo MTBC isolates were genotyped using spoligotyping as well as MIRU-VNTR at the strain level, as presented in Tables 3 and 4. The discriminatory power of spoligotying and 24-loci MIRU-VNTR were 0.9700 and 0.9995, respectively. The analysis of spoligotypes resulted in 66 different patterns of which 24 clustered and 42 were unique. MIRU-VNTR analysis resulted in 149 different patterns of which six clustered and 143 were unique. The clustering rates were 57.7% ((114–24)/156) for spoligotying and 3.9% ((12–6)/155) for the MIRU-VNTR analysis. The RTIs for South Omo were calculated to be 57.7% based on spoligotyping and 3.9% based on MIRU-VNTR data.

**Table 3:** Discriminatory power of genotyping methods

Typing Method	Different patterns (N) (%)	Clusters (N)	Cluster size (N)	Clustered isolates (N) (%)	Unique isolates (N) (%)	Clustering rate %	HGDI
Spoligotyping	66/156 (42.3)	24	2–17	114 (73.1)	42 (26.9)	57.7	0.97
MIRU-VNTR-24	149/155 (96.1)	6	2	12 (7.7)	143 (92.3)	3.9	0.9995
MIRU-VNTR −15	148/155 (95.5)	7	2	14 (9.0)	141 (91.0)	4.5	0.9994
MIRU-VNTR-12	102/155 (65.8)	23	2–8	76 (49.0)	79 (51.0)	34.2	0.9897
Spoligotyping + MIRU-VNTR	155/155 (100)	0	0	0	155 (100)	100	1

*HGDI* Hunter Gaston Discrimination index

**Table 4:**
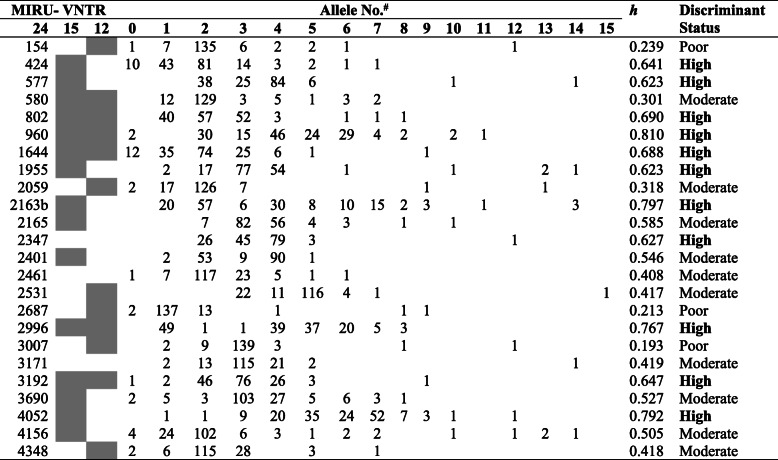
Occurrence of MIRU-VNTR alleles and allelic diversity

The MIRU-VNTR locus in the Mtb genome; ^#^ the number of alleles pertains to the frequency with which a distinct repeat unit was identified among the 155 isolates. High discriminant (h > 0.6), moderate discriminant (0.3 < = h < =0.6) and poor discriminant (h < 0.3) [34]. Shaded boxes show alleles used for the 15-loci MIRU-VNTR and the 12-loci MIRU-VNTR methods

Allelic diversities (*h*) for each MIRU-VNTR locus are presented in Table 4. MIRU-VNTR loci are classified based on their allelic diversity and ability to discriminate among the isolates. Eleven MIRU-VNTR loci (424, 577, 802, 960, 1644, 1955, 2163b, 2347, 2996, 3192 and 4052) were highly discriminatory while 10 MIRU-VNTR loci (580, 2059, 2165, 2401, 2461, 2531, 3171, 3690, 4156 and 4348) were moderately discriminatory. The remaining three loci showed poor discriminatory values for the isolates under study. However, 11-loci are considered as moderately discriminatory according to newly proposed discriminatory values [27].

### Spoligotyping and MIRU-VNTR based identification of lineages and sub-lineages

**Fig. 1:**
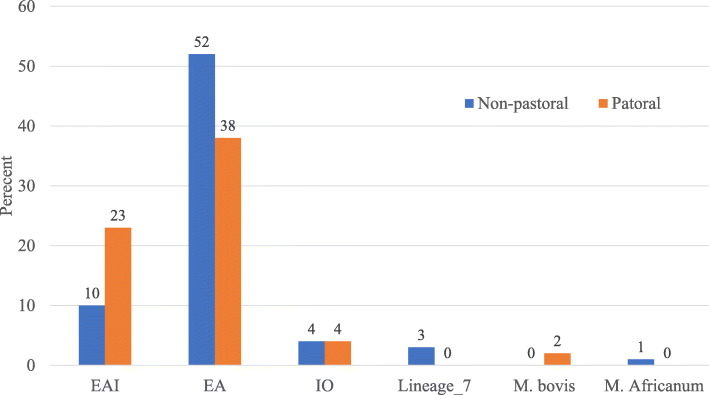
Major lineage prevalence in pastoral and non-pastoral areas of South Omo, southern Ethiopia. Abbreviations: EA, Euro-American; EAI, East-African-Indian; IO, IndoOceanic; M. afri., *M. africanum.* The numbers on top of the bars indicate absolute number of Major lineages

The results of spoligotyping and MIRU-VNTR are presented in Tables 5 and 6. According to spoliogotyping results 76.3% (119/156) of the isolates belonged to 36 shared types (SIT numbers). The remaining, about one-qaurter of the isolates (37/156) were orphan strains. Euro-American (EA) was the most prevalent lineage with 67.3% (105/156) followed by East-African-Indian (EAI) with 22.4% (35/156) and Indo-Oceanic (IO) with 6.4% (10/156) of the isolates. Interestingly, EA was more prevalent in non-pastoral areas while EAI was more prevalent in pastoral areas (Fig. 1). Two *M. bovis* isolates were identified in pastoral areas.

**Table 5:**
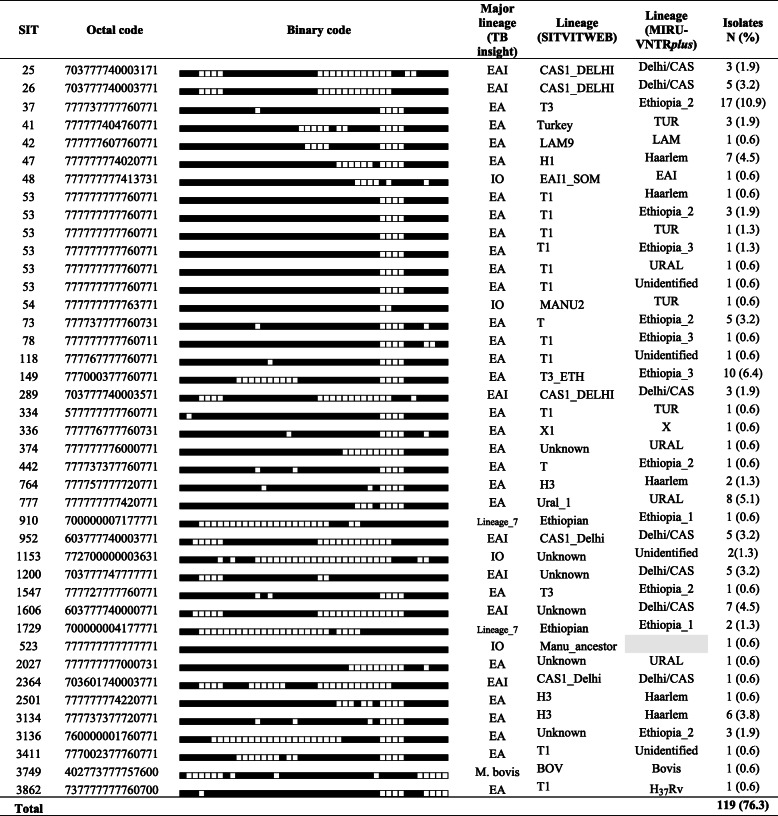
Major lineages and lineages of MTBC strains concordant with existing SIT numbers

Abbreviations: *EA* Euro-American, *EAI* East-African-Indian, *IO* Indo-oceanic

**Table 6:**
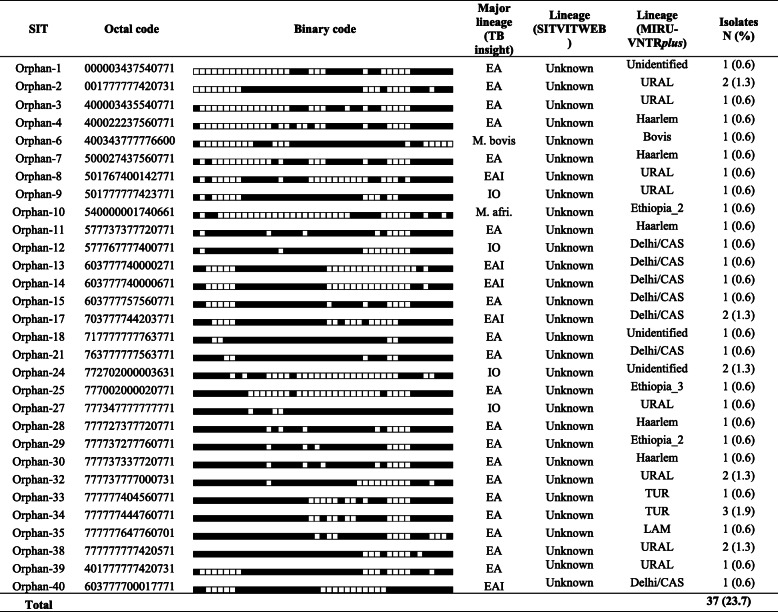
Major lineages and lineages of orphan MTBC strains

*Abbreviations CBN* Conformal Bayesian network, *EA* Euro-American, *EAI* East-African-Indian, *IO* IndoOceanic, *M. afri*., *M. africanum*

**Table 7:** Association of different factors with clustering of *M. tuberculosis* complex

Characteristics	Total N (%)	Clustered N (%)	COR (95% CI)	***P*** value	AOR (95% CI)	***P*** value
Sex						
Female	64 (41.0)	46 (71.9)	1.00		NA	
Male	92 (59.0)	68 (73.9)	1.11 (0.54–2.27)	0.778		
Age in years						
15–29	70 (44.9)	55 (78.6)	1.00		NA	
30–44	59 (37.8)	41 (69.5)	0.62 (0.28–1.38)	0.241		
> =45	27 (17.3)	18 (66.7)	0.55 (0.20–1.46)	0.227		
TB Type						
PTB	130 (83.3)	92 (70.8)	1.00		NA	
EPTB	26 (16.7)	22 (84.6)	2.27 (0.73–7.04)	0.155		
Medical history						
New cases	148 (94.9)	107 (72.3)	1.00		NA	
Retreat. Cases	8 (5.1)	7 (87.5)	2.68 (0.32–22.48)	0.363		
Residential area						
Non-pastoral	70 (51.1)	48 (68.6)	1.00		1	
Pastoral	67 (48.9)	56 (83.6)	**2.33 (1.03–5.30)**	**0.043**	2.29 (0.82–6.35)	0.112
Major lineages^a^						
EAI	35 (22.9)	30 (85.7)	1.00		1.00	
EA	105 (68.6)	78 (74.3)	0.48 (0.17–1.37)	0.170	0.59 (0.16–2.22)	0.439
IO	10 (6.5)	4 (40.0)	**0.11 (0.02–0.54)**	**< 0.006**	0.24 (0.03–1.83)	0.169
Lineage_7	3 (2.0)	2 (66.7)	0.33 (0.03–4.40)	0.404	0.22 (0.01–3.50)	0.110
SIT status						
Shared type	119 (76.3)	101 (84.9)	1.00		1.00	
Orphan	37 (23.7)	13 (35.1)	**0.10 (0.04–0.22)**	**< 0.001**	**0.09 (0.04–0.25)**	**< 0.001**
Lineage^a^						
Delhi/CAS	37 (26.6)	30 (81.1)	1.00		NA	
Haarlem	22 (15.8)	16 (72.7)	0.62 (0.18–2.17)	0.456		
URAL	22 (15.8)	15 (68.2)	0.50 (0.15–1.69)	0.264		
Ethiopia_2	32 (23.0)	28 (87.5)	1.63 (0.43–6.19)	0.470		
TUR	10 (7.2)	7 (70.0)	0.54 (0.11–2.65)	0.452		
Ethiopia_3	13 (9.4)	11 (84.6)	1.28 (0.23–7.14)	0.776		
Ethiopia_1	3 (2.2)	2 (66.7)	0.47 (0.04–5.90)	0.556		

^a^Sub-categories having only singletons or clustered (based on strain clustering data using spoligotype patterns) were excluded from the regression analysis; NA: Not applicable; some abbreviations sued here were defined in the legend of Table 2

According to SITVIT analysis, the identified lineages were T 30.1% (47/156), CAS1_Delhi 10.9% (17/156), Haarlem 10.3% (16/156), Ethiopian 1.9% (3/156), Turkey 1.9 (3/156), MANU 1.3% (2/156), LAM 0.6% (1/156), EAI1_SOM 0.6% (1/156), BOV 0.6% (1/156) and X 0.6% (1/156). Almost one-third of isolates (56/156) were not registered in updated version of SITVITWEB database (SITVIT2). According to MIRU-VNTR*plus* analysis result (S1 Fig, Table 5 and Table 6), the identified lineages were Delhi/CAS 23.9% (37/155), Ethiopia_2 20.6% (32/155), Haarlem and URAL 14.2% (22/155) each, Ethiopia_3 8.4% (13/155), TUR 6.5% (10/155), Ethiopia_1 1.9% (3/155), Bovis and LAM 1.3% (2/155) each, and EAI, X and Ethiopia H_37_Rv-like strain 0.6% (1/155) each. Around 6% of the isolates were not assigned into lineages using MIRU-VNTRplus database.

### Minimum spanning tree (MST) analysis

**Fig. 2:**
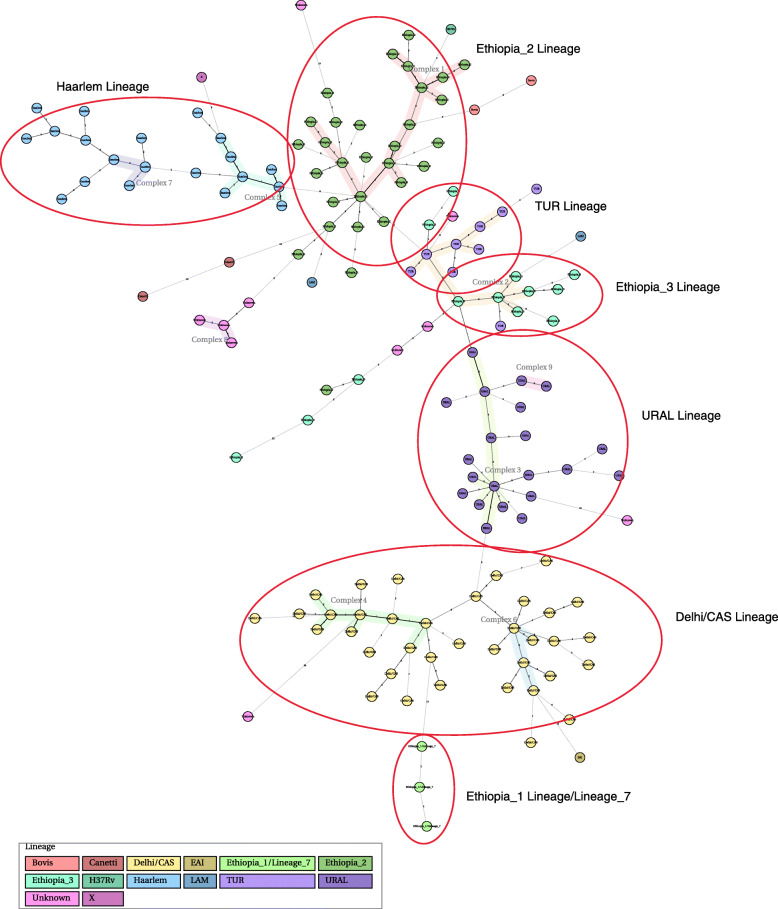
Minimum spanning tree (MST) based on the 24 MIRU-VNTR data of 155 strains isolated from South Omo, Southern Ethiopia. Sub-lineages were colored differently. Each individual pattern is represented by a circle. The length of the branch represents the distance between patterns. A maximum locus difference within a clonal complex of MIRU-VNTR types was a double locus variation (DLV). *M. canetti* from MIRU-VNTRplus database

MST shows the relationship among strains and lineages based on MIRU-VNTR loci variant count. Five well-defined branches (Complex 1, 2, 3, 4 and 5) and four additional sub-branches (Complex 6, 7, 8 and 9) expanding from Complex 1 are featured in Fig. 2. Each complex largely consisted of distinct sub-lineages. Part of Complex 1 are the Ethiopia_2 sub-lineage and the additional sub-lineages H37Rv, Bovis, unidentified strains, and *M. canetti* (used as a reference). Complexes 2, 3, 4 and 5 are enriched in Ethiopian_3 and TUR, URAL, Delhi/CAS and Haarlem strains, respectively.

### Factors associated with strain clustering

Clustering of *M. tuberculosis* strains association with different factors were analyzed using spoligotyping pattern. Clustering was significantly associated with the residential area, the major lineage type and the SIT status before adjusted to confounding factors (Table 7). However, only being part of the orphan group compared to shared type (AOR: 0.19, 95% CI: 0.04–0.25) was significantly associated with reduced odds of clustering in the area under study after adjustment with confounding factors.

## Discussion

This study was the first of its kind to analyze the MTBC population structure and transmission dynamics in the South Omo Zone, southern Ethiopia. The study included PTB and EPTB patients, and identified highly diverse lineages. The clustering rate/RTI was low in the study area. Logistic regression analysis showed that clustering of strains was associated with SIT status.

Health facilities other than JGH in the study area are in range of 14 to 250 km from Jinka town where JGH and the Regional Laboratory are located. Due to feasibility, samples were stored in health facilities at -20 °C from a week to 3 weeks. The variation in culture recovery rate of MTBC isolates in this study possibly associated with sample storage conditions. There was continuous electric interruption in the Zone which affects the storage temperature which could compromise the viability of MTBC in the sample. JGH had its own backup generator that might be the reason for better recovery rate for samples from JGH. In connection, the overall culture recovery rate in this study is less than previous studies in Ethiopia [47, 48].

In contrast to previous reports in the study area [8, 41], the number of EPTB cases in this study was low. From personal observations, the low number of EPTB cases in this study might be due to lack of skilled pathologist to take FNA samples whereas in previous studies relied on clinical symptoms. Spoligotyping and MIRU-VNTR are recommended methods for the profiling of MTBC isolates [23, 35]. Both genotyping methods in this study were in range of highly discriminant [27, 34]. MIRU-VNTR has higher discriminatory value than spoligotyping as shown here and in earlier studies [1, 9, 36, 37].

Spoligotyping of South Omo MTBC isolates resulted in a clustering rate of 57.7%%. This rate agrees with a previous study in Gambella, Southwest Ethiopia [3] which is geographically proximate to the present study site. The rate is lower than a national survey [19] and that of studies in Addis Ababa [26], Northwest Ethiopia [37], Eastern Ethiopia [7], and central Ethiopia [6]. But it was a study in Western Ethiopia [16]. The MIRU-VNTR clustering rate was 3.9%. The rate is lower than other studies in Ethiopia [1, 9, 36, 37, 43] and higher than a Chinese study [15]. Such variability in clustering rate among studies could be due to differences in geography, population density, ethnicity and socio-economic diversity [31]. The low clustering rate in our study could also be associated with low culture recovery rate of samples which make potential isolates from the study population not to be genotyped and/or presence of low TB transmission in South Omo due to geographic expanse which disfavor the transmission as a result of very less crowdedness in the community.

Most MIRU-VNTR alleles in this study were highly and moderately discriminant based on the allelic diversity (*h*) which is an indirect indicator of the sample representativeness of the study population [34]. Values of *h* more than 0.8 and less than 0.1 are unsuitable for genotyping [30]. In the present study, all MIRU-VNTR loci were suitable for genotyping of the isolates except locus 960 with an *h* value of 0.810.

This study identified six major lineages (EA, EAI, IO, lineage_7, *M. bovis* and *M. africanum*). Four isolates were identified using TB insight database as *M. africanum* which is known to be localized in West Africa. However, three of the four isolates were re-identified using updated version of SITVITWEB as Ethiopian and considered as Lineage_7/Ethiopia_1 [9, 17, 37] but one isolate remained unknown. The population in the area is endogenous which make the plausibility of an unknown isolate to be *M. africanum* less probable. In general, this might imply the need of updating TB Insight database with newly generated MTBC data from the horn of Africa, particularly Ethiopia.

EA is the most dominant lineage in the world [32], ranging in Ethiopia from 32.5% near the border to South Sudan [3] to 86.8% in central Ethiopia [6]. This might highlight the introduction of EA lineages from abroad through the capital city and their expansion to the peripheral areas. In addition, the existence of EA in high number in contrast to Ethiopian lineage, Lineage_7, in the country might indicate the high transmission ability of EA. The contribution of *M. bovis* for TB was low which is supported by data from other studies [7, 17]. While larger clinical studies are needed, our data suggested that the role of *M. bovis* as a causative agent of TB in pastoral area presumably linked to contact with infected cattle and, consumption of raw milk and meat [5].

According to the updated version of SITVITWEB database, the T lineage was predominant in this study. This lineage accommodates MTBC strains which do not have phylogeographic specificity [12]. Among the T sub-lineages, T3 (42.2%) was the most dominant one in this study. It is also called Ethiopia_2 [9] which is followed by T3-ETH, also called Ethiopia_3 [9]. These isolates supposed to be phylogeographically specific to Ethiopia including well defined Ethiopian lineage also called lineage_7 [17]. The CAS1_Delhi lineage was the second predominant lineage in the study area which was followed by Haarlem. The Haarlem is believed to descend from the European continent [12]. The predominance of the T lineage in Ethiopia and the CAS lineage in Tanzania [25] and Kenya [29] supports the notion of enrichment of MTBC strains in certain geographies. The Turkey lineage was present in the study area which is believed to be specific to Turkey [45]. This might be associated with presence of Turkey investors in the South Omo. In addition, Ural_1, Manu, Bov, EAI_SOM, and X lineages were identified in less frequency in the area. It is plausible that the observed phylogeographic diversity has linked to considerable international tourism in South Omo, Ethiopia.

When we look at MIRU-VNTR*plus* based lineage in the present study, Delhi/CAS was the predominat one which is in agreement with previous studies in Ethiopia [9, 36, 37, 43]. Ethiopia_2 is the predominant Ethiopia specific lineage followed by the Ethiopia_3 and lineage_7, similar to a previous study in geographic proximity, Southwestern Ethiopia [36]. But in studies at far distance, in Northwestern Ethiopia [9, 43], the predominant lineage was lineage_7 followed by the Ethiopia_3 and Ethiopia_2 lineages. These findings indicate that the distribution of Ethiopia specific lineages differ moderately from area to area within the country localities. This information is useful for the country’s TB Control Program. Almost 6% of isolates in this study were not assigned into lineages which requires further study and introduction into the genotype database. The relationship among lineages in the MST based on MIRU-VNTR loci was in agreement with similar studies conducted in Ethiopia [9, 37].

Based on the generated data from spoligotyping and MIRU-VNTR, it is possible to say that these two methods can complement each other. But they have different precisions. For instance, MIRU-VNTRplus can identify 37 Delhi/CAS, 22 Haarlem and 22 Ural lineages whereas SITVITWEB can only identify 17 CAS1_Delhi, 16 Haarlem and eight Ural_1. This differences probably associated with the algorithm used by such databases. Finally, from all isolates of South Omo in this study, MIRU-VNTRplus and SITVITWEB didn’t assign nine and 56 isolates into lineages, respectively.

The multivariate logistic regression analysis in this study showed none of the variables had association with strain clustering except SIT shared status. Orphan strains were less likely to cluster compared to shared strains which implies that shared strains have higher transmission rate compared to orphan strains in the study area.

We contend that the number of genotyped isolates is sufficient for a primary representation of South Omo’s MTBC population structure, assessment of clustering rates and RTIs. However, having less culture recovery rate from samples other than JGH in this study limited our ability to identify clusters and RTIs more comprehensively.

## Conclusion

The MTBC strains derived from TB patients in South Omo were highly diverse while the RTI was low in this marginalized region, as compared to other studies in high TB burden countries. Nonetheless, the genotyping data are useful as an input to map the population structure of MTBC in Ethiopia and to the TB Control Program in the pastoral region.

## Supplementary information

**Additional file 1.** Phylogenetic tree for 155 isolates was constructed based on 24 locus MIRU-VNTR. The dendrogram was calculated using neighbor-joining clustering algorithms.

## Data Availability

The data generated or analysed during this study are not publicly available due to it may compromise patient anonymity but are available from the corresponding author on reasonable request.

## Abbreviations

AAU: Addis Ababa University
ALIPB: Aklilu Lemma Institute of Pathobiology
AOR: Adjusted odds ratio
CI: Confidence interval
COR: Crude odds ratio
DOTS: Direct observed treatment short course
EA: Euro-American
EAI: East-African-Indian
EPTB: Extrapulmonary tuberculosis
FNA: Fine needle aspirate
HGDI: Hunter-Gaston Discriminatory Index
JCVI: J. Craig Venter Institute
JRL: Jinka Regional Laboratory
IO: Indo-Oceanic
LJ: Lowenstein Jensen
MIRU-VNTR: Mycobacterial interspersed repetitive unit-variable number of tandem repeat
MTBC: *Mycobacterium tuberculosis* complex
MST: Minimum spanning tree
PCR: Polymerase chain reaction
PTB: Pulmonary tuberculosis
RD: Region difference
RTI: Recent transmission index
TB: Pulmonary tuberculosis
ZN: Ziehl-Neelsen
