# Supplementary material for: Molecular epidemiology of clinical Mycobacterium tuberculosis complex isolates in South Omo, Southern Ethiopia
Source: BMC Infect Dis. 2020 Oct 13;20:750. doi: 10.1186/s12879-020-05394-9 (PMC7557052; doi:10.1186/s12879-020-05394-9)
Supplement: Supplementary file 1 — Additional file 1. Phylogenetic tree for 155 isolates was constructed based on 24 locus MIRU-VNTR. The dendrogram was calculated using neighbor-joining clustering algorithms. [file 12879_2020_5394_MOESM1_ESM.pdf]

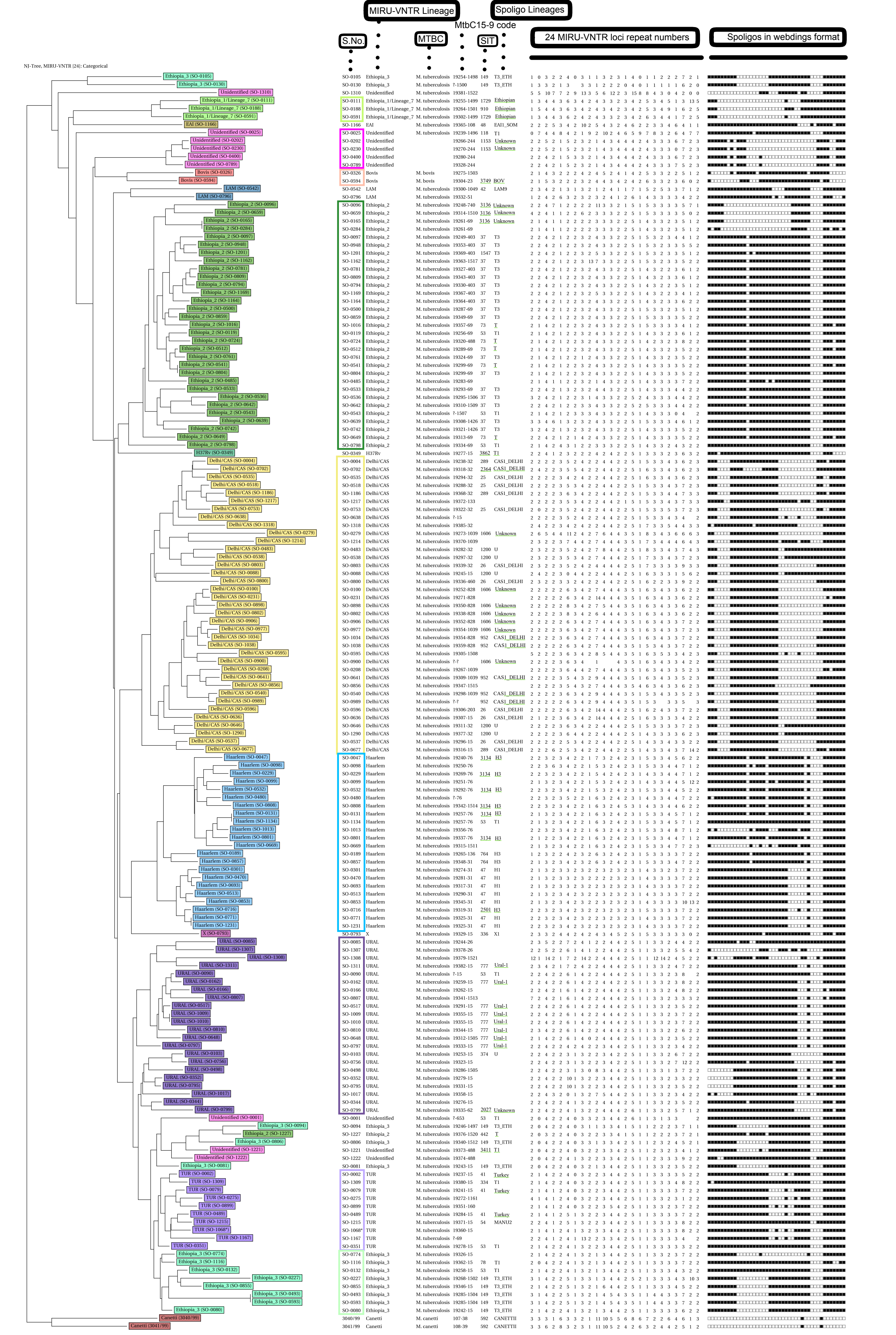

S1- Phylogenetic tree for 155 isolates was constructed based on 24 locus MIRU-VNTR. The dendrogram was calculated using neighbor-joining clustering algorithms.
